# Neuronal Damage Induced by Perinatal Asphyxia Is Attenuated by Postinjury Glutaredoxin-2 Administration

**DOI:** 10.1155/2017/4162465

**Published:** 2017-06-15

**Authors:** Juan Ignacio Romero, Mariana Inés Holubiec, Tamara Logica Tornatore, Stéphanie Rivière, Eva-Maria Hanschmann, Rodolfo Alberto Kölliker-Frers, Julia Tau, Eduardo Blanco, Pablo Galeano, Fernando Rodríguez de Fonseca, Christopher Horst Lillig, Francisco Capani

**Affiliations:** ^1^Instituto de Investigaciones Cardiológicas “Prof. Dr. Alberto C. Taquini” (ININCA), Facultad de Medicina, UBA-CONICET, Marcelo T. de Alvear 2270, C1122AAJ, Ciudad de Buenos Aires, Argentina; ^2^Department of Neurology, Medical Faculty, Heinrich-Heine-University, Düsseldorf, Germany; ^3^Institute for Medical Biochemistry and Molecular Biology, Universitätsmedizin Greifswald, Ernst-Moritz-Arndt-Universität Greifswald, 17475 Greifswald, Germany; ^4^Laboratory of Ocular Investigation, Department of Pathology, School of Medicine, University of Buenos Aires, Buenos Aires, Argentina; ^5^Departament de Pedagogia i Psicologia, Facultat d′'Educació, Psicologia i Treball Social, Universitat de Lleida, Avda. de l'Estudi General 4, 25001 Lleida, Spain; ^6^Fundación Instituto Leloir, Av. Patricias Argentinas 435, C1405BWE, Ciudad Autónoma de Buenos Aires, Argentina; ^7^Unidad de Gestión Clínica de Salud Mental, Instituto de Investigación Biomédica de Málaga, Hospital Regional Universitario de Málaga, Universidad de Málaga, Avda. Carlos Haya 82, 29010 Málaga, Spain; ^8^Departamento de Biología, Universidad Argentina JF Kennedy, Buenos Aires, Argentina; ^9^Investigador Asociado, Universidad Autónoma de Chile, Santiago, Chile

## Abstract

The general disruption of redox signaling following an ischemia-reperfusion episode has been proposed as a crucial component in neuronal death and consequently brain damage. Thioredoxin (Trx) family proteins control redox reactions and ensure protein regulation via specific, oxidative posttranslational modifications as part of cellular signaling processes. Trx proteins function in the manifestation, progression, and recovery following hypoxic/ischemic damage. Here, we analyzed the neuroprotective effects of postinjury, exogenous administration of Grx2 and Trx1 in a neonatal hypoxia/ischemia model. P7 Sprague-Dawley rats were subjected to right common carotid ligation or sham surgery, followed by an exposure to nitrogen. 1 h later, animals were injected i.p. with saline solution, 10 mg/kg recombinant Grx2 or Trx1, and euthanized 72 h postinjury. Results showed that Grx2 administration, and to some extent Trx1, attenuated part of the neuronal damage associated with a perinatal hypoxic/ischemic damage, such as glutamate excitotoxicity, axonal integrity, and astrogliosis. Moreover, these treatments also prevented some of the consequences of the induced neural injury, such as the delay of neurobehavioral development. To our knowledge, this is the first study demonstrating neuroprotective effects of recombinant Trx proteins on the outcome of neonatal hypoxia/ischemia, implying clinical potential as neuroprotective agents that might counteract neonatal hypoxia/ischemia injury.

## 1. Introduction

One of the main causes of neonatal death and neurological deficits in children is an insufficient oxygen supply during birth, known as perinatal asphyxia (PA). The central nervous system (CNS), particularly the brain, is damaged as a result of the combination of hypoxia, blood flow reduction (ischemia), and reoxygenation [1]. This pathology is associated with an increase in the levels of glutamate [2] and the consequent release of nitric oxide (NO) [3, 4]. Moreover, an increase of the superoxide anion radical (O2-·) which can rapidly react with NO to form peroxynitrite anions (ONOO-) has been described [5]. The excess of these molecules and the unspecific damage to various biomolecules have been described as oxidative stress in the 80th and have been linked to cell death, inflammation and might even explain the high mortality risk. Nowadays, the definition of oxidative stress has been paraphrased, acknowledging the function of different reactive species in physiological signaling cascades as well as their dysregulation in pathological conditions [6].

Redox signaling is regulated by proteins of the thioredoxin (Trx) family such as Trxs, glutaredoxins (Grxs) and peroxiredoxins (Prxs), that use thiol groups of cysteinyl residues within their active site motifs to catalyze for example thiol-disulfide exchange reactions [7, 8] (for an overview, see [6]). These redox proteins show tissue- and cell type-specific distributions. For instance, our work demonstrates specific differences in terms of protein abundance and distribution in the murine CNS [9, 10]. Trx family proteins possess the representative common structural motif, known as the Trx fold [11]. Trxs and Grxs are small oxidoreductases that contain the characteristic and highly conserved dithiol Cys-X-X-Cys motif in their active site that is essential for the reduction of specific disulfide bonds via the dithiol mechanism. Moreover, Trxs can catalyze the de-/nitrosylation of substrates and Grxs can catalyze the de-/glutathionylation of target proteins via the monothiol mechanism (reviewed in [12]). Trxs and Grxs can reduce Prxs, which are not only highly abundant peroxidases but rather regulators of the levels of the second messenger hydrogen peroxide and therefore essential for redox signaling [6, 12].

Interestingly, these proteins were shown to be altered in terms of expression, cellular distribution, and/or activity in various disorders. Also in diseases linked to hypoxia/ischemia, Trxs, Grxs, and Prxs and thereby cellular signaling within distinct cell types and tissues are significantly altered (for an overview, see [6]). Following cerebral ischemia induced by middle cerebral artery occlusion, Trx1 was reduced in ischemic areas and increased in perifocal ischemic regions [13]. Trx1 was induced in hippocampal glial cells during reperfusion following a transient cerebral ischemia in gerbils [14]. Transgenic mice overexpressing human Trx1 showed attenuated ischemic neuronal injury and significantly smaller infarct sizes when subjected to focal cerebral ischemia [15]. Moreover, Trx1 overexpression was shown to protect mice from neuronal apoptosis following mild focal ischemia [16]. In accordance with this evidence, the knock-down of Trx1 led to more pronounced neurological dysfunction, brain infarct size, brain edema, and overall peroxidation [17], as well as exacerbated apoptosis of neurons, behavioral deficits, and mortality [18]. Interestingly, the overexpression of the mitochondrial Trx2 attenuated ROS-induced TNF-*α* expression and subsequent NF-*κ*B activation and apoptosis [19]. Additionally, the protein levels of the cytosolic Grx1 were diminished after middle cerebral artery occlusion, correlating with neuronal damage [20]. Moreover, the overexpression of Prx2 was shown to protect cortical neuronal cell cultures from oxidative and ischemic damage [21]. Furthermore, exogenous administration of human Trx1 was shown to be able to pass the blood-brain barrier and exert a positive effect on neurogenesis promotion and cognitive recovery following cerebral ischemia in adult mice [22].

We have recently shown an increase in the hippocampal protein levels of Trx1 and Grx2 following a neonatal hypoxic/ischemic event. The specific knock-down of these proteins in a neuron-like cell model allowed us to characterize the importance of these proteins in neuronal differentiation and maturation after a hypoxic/ischemic reperfusion event. Particularly, both Grx2 and Trx1 seem to protect neuronal cells from hypoxia-induced damage, while Trx1 knock-down decreases cellular proliferation and viability. Moreover, the absence of either Grx2 or Trx1 following hypoxia and reoxygenation triggers differentiation into a glial-like cell type [23]. Interestingly, it was shown before in zebra fish that the loss of glutaredoxin leads to neuronal apoptosis and loss of an axonal scaffold, making Grx an essential protein for embryonic brain development [24].

Several potential neuroprotective agents have been used in the past to ameliorate the hypoxia/ischemia-induced damage in the CNS; however, neither of them has shown to be effective against hypoxia/ischemia-induced damage in the CNS. Here, we demonstrate the neuroprotective effects of exogenously administered, recombinantly expressed Grx2 and Trx1 (particularly in the hippocampus) in an animal model of neonatal hypoxia/ischemia, implying potential new therapeutic strategies.

## 2. Experimental Procedures

### 2.1. Animals

All experiments were conducted according to the principles of the guide for the care and use of laboratory animals (NIH publication no. 80-23, revised 1996) and approved by the institutional animal care and use committee at the University of Buenos Aires (School of Medicine). All efforts were made to reduce the number of animals used and to minimize suffering. Pregnant rats were obtained from the “School of Veterinary Sciences” central *vivarium* at the University of Buenos Aires. All animals were kept in a temperature (21 ± 2°C) and humidity (65 ± 5%) controlled environment on a 12 h light/dark cycle. Animals had ad libitum access to food (Purina chow) and tap water.

### 2.2. Model for Common Carotid Artery Ligation and Treatment

The model for common carotid artery ligation used in this study has been previously developed and validated [23, 25]. P7 male Sprague-Dawley rats were anesthetized (40 mg/kg ketamine and 4 mg/kg xylazine) and placed on a heat plate to keep their body temperature at constant 37°C. The right common carotid artery (CCA) was exposed through an incision on the neck and was then isolated and permanently ligated with a 6-0 surgical silk thread (carotid group *n* = 27). Afterwards, the wound was closed and pups were returned to their dams for 4-5 h to recover. Subsequently, the animals were subjected to a 100% nitrogen environment at 37°C for 3 minutes to induce anoxia. Sham-operated rats (sham group *n* = 27) had their right CCA exposed but not ligated, and no nitrogen was applied. One hour after nitrogen exposure, animals were injected intraperitoneally (i.p.) with either saline solution (vehicle group *n* = 9), 10 mg/kg of recombinantly expressed and purified human Grx2 (Grx2 group *n* = 9), or 10 mg/kg of recombinantly expressed and purified human Trx1 (Trx1 group *n* = 9). At 72 h postinjury (postnatal day 10 (P10)), animals were sacrificed and their blood plasma and brains were collected for further analysis. The cloning, recombinant expression, and purification of hGrx2 and hTrx1 are described in Lundberg et al. [26] and Godoy et al. [9] and were produced in Dr Lillig's laboratory. All human recombinant Grx2 and Trx1 were processed to exchange the original buffer for phosphate-buffered saline (PBS) and increase protein concentration. Before injection, each concentrate was diluted (at least 10-fold) with sterile saline solution in order to reach a 10 mg/kg dosage.

### 2.3. Western Blotting

Western blot analysis was performed as previously described in Romero et al. [23]. Animals were euthanized by decapitation, and brains were dissected, homogenized in ice-cold lysis buffer (10 mM Tris/HCl, pH 7.4, 10 mM NaCl, 3 mM MgCl_2_, 0.1% NP-40, protease inhibitors), and fast frozen in liquid nitrogen. The tissues were then thawn on ice and centrifuged at 13000 rpm for 15 min at 4°C. The supernatants were analyzed for total protein concentration using Bradford solution (Biorad, Munich, Germany) in 96-well plates with bovine serum albumin (BSA) as standard. 10–20 *μ*g of total protein were diluted in sample buffer (0.3 M Tris/HCl, pH 7, 50% glycerol, 5% SDS, 1 mM EDTA, 0.1% bromphenol blue) and were treated with 100 mM DTT for 30 min at room temperature followed by 10 min at 94°C. Samples were subjected to SDS–PAGE using the Mini-Protean TGX stain-free 4–20% precast gels (Biorad) and were transferred to PVDF membranes according to the manufacturer's instructions.

Membranes were blocked with 5% nonfat milk powder and 1% BSA in Tris-buffered saline containing 0.05% Tween 20 and incubated with specific primary antibodies at 4°C overnight. Antigen-antibody complexes were stained using horseradish peroxidase- (HRP-) coupled antibodies (Biorad, Richmond, CA, USA) and the enhanced chemiluminescence method. Luminescence was recorded using a gel documentation system (ChemiDoc™ XRS+ System). The total protein amount in each lane was quantified using the stain-free technology of Biorad and was used for normalization of the blotting data obtained from densitometric analysis [27, 28]. Antibodies detecting HSP70 (4873S, dil 1 : 1000) and PSD95 (ab18258, dil 1 : 1000) were purchased from Cell Signaling Technology (Danvers, USA) and Abcam (Cambridge, USA), respectively.

### 2.4. ELISA

A specific sandwich ELISA kit (NS170, Chemicon International, USA-Canada) was used to quantify the heavy chain of phosphorylated neurofilaments (pNF-H) according to the manufacturer's instructions. Briefly, a 96-well immunoplate precoated with chicken anti-pNF-H polyclonal antibody was used to capture pNF-H in plasma samples, as well as specific standards with known pNF-H concentrations. Captured pNF-H was then detected by a rabbit anti-pNF-H polyclonal antibody (1 : 100) and followed by an alkaline phosphatase-conjugated goat anti-rabbit polyclonal antibody. After the addition of the substrate pNPP (p-nitrophenyl phosphatase), the amount of pNF-H was determined by absorbance at 405 nm.

### 2.5. Immunohistochemistry and Cell Counting

Immunohistochemistry was performed as previously described in Romero et al. [23]. Animals were anesthetized with 28% (*w*/*v*) chloral hydrate, 0.1 ml/100 g of body weight, and intracardially perfused with 4% paraformaldehyde (Sigma-Aldrich, St. Louis, MO, USA) freshly prepared in 0.1 M phosphate buffer, pH 7.4. Brains were dissected and postfixed in the same solution for 2 h. Coronal brain sections (4 *μ*m thick) were cut using a Leica sliding microtome and then recovered for light microscopy studies. Prior to the staining, sections were incubated in 3% hydrogen peroxide for 10 min to quench endogenous peroxidases. After three washing steps in PBS, nonspecific antibody binding sites were blocked with 10% goat serum (Invitrogen Corporation, Camarillo, CA, USA) in PBS and sections were incubated overnight with anti-GFAP rabbit polyclonal antibody (1 : 500, Z0334, Dako, Germany) at 4°C. Then, sections were washed three times with PBS and subsequently incubated with a goat anti-rabbit biotinylated secondary antibody (1 : 500, BA-1000, Vector Laboratories Inc., USA) for 60 min at room temperature. The streptavidin/HRP detection system (P0397, Dako, Germany) was used for antigen staining according to the manufacturer's recommendations. Sections were incubated with the substrate diaminobenzidine (11718096001, Roche Life Science, USA) for 5 min at room temperature. Finally, sections were dehydrated and were mounted with Canada balsam (Sigma-Aldrich, St. Louis, MO, USA). Sections without incubation with the primary antibody were used as a control to verify the specificity of the secondary antibody. Samples were examined by light microscopy using a Leitz Laborlux S microscope (Heidelberg, Germany) equipped with a CCD video camera (Canon). Images were analyzed and compiled using Adobe Photoshop 11.0 CS4. Note that for protein staining, all samples (both sham and carotid groups) were processed together in the same batch, using the same antibody dilutions and the same time for DAB development.

Cell counting analysis was carried out in 5 to 6 coronal sections obtained from −3.14 mm to −4.30 mm Bregma levels (dorsal hippocampus) [29], for a total of 4 animals per group. In every section, the number of GFAP positive cells was quantified in both hemispheres and averaged. A mean was calculated for each animal and used for subsequent statistical analysis. We determined the cell number per area using the “Cell Counter” Image J 1.38X plugin tool (NIH, USA). In sections stained with GFAP containing *Cornu Ammonis* 1 (CA1), we set 0.02 mm^2^ squares along the *Stratum pyramidale* (sp) of CA1 in such a manner that the whole area was represented. Subsequently, we manually determined the number of cells in each square and calculated the number of cells per mm^2^. All quantifications were performed in a blind approach.

### 2.6. Neurobehavioral Studies

Neurobehavioral development studies were carried out from the first day after hypoxia-ischemia induction (P8), till the last animal showed the appearance or disappearance of the analyzed reflex (~P19). Animals were evaluated every day at the same time (10 a.m.) for approximately 3 hours each time. All scoring was performed blindly by two observers. In order to evaluate the possible effect of the treatment in the neurobehavioral development of the animals, pups were subjected to a series of tests previously described by Kiss et al. [30], Shahrokhi et al. [31], and Giriko et al. [32], which assess the manifestation of different reflexes such as surface righting reflex, negative geotaxis, crossed extensor reflex, ear and eyelid twitching, and auditory startle. Additionally, ear unfolding and eye opening were assessed as a measurement of physical development.

### 2.7. Statistical Analysis

Band intensities obtained from Western blots were quantified using GelPro 3.1 and were expressed as percentage of the control levels (sham-operated rats injected with saline solution). Total protein amount, visualized using the stain-free technology of Biorad was quantified using the ImageLab 5.0 software (Biorad). Bar diagrams depict the mean of four independent quantifications for each experiment, consisting of sham-operated (sham-veh, *n* = 5; sham-Grx2, *n* = 5; and sham-Trx1, *n* = 5) and ischemic (carotid-veh, *n* = 5; carotid-Grx2, *n* = 5; and carotid-Trx1, *n* = 5) animals + SEM, correlated to total protein. Two-way ANOVA tests with condition (sham, carotid) and treatment (vehicle, Grx2, and Trx1) as between-subject factors, followed by Tukey's post hoc tests, were employed to analyze the biochemical parameters and protein levels. For the neurobehavioral analysis, bar diagrams depict the mean of the day of appearance of the assessed reflexes for each group, consisting of sham-operated (sham-veh, *n* = 10; sham-Grx2, *n* = 10; and sham-Trx1, *n* = 10) and ischemic (carotid-veh, *n* = 10; carotid-Grx2, *n* = 10; and carotid-Trx1, *n* = 10) animals + SEM. Kruskal-Wallis tests, followed by Mann–Whitney tests for pair-wise multiple comparisons, were employed to analyze neurodevelopmental parameters. In all cases, the level of significance was set up at 5%. All analyses were performed using SPSS 15.0 (Chicago, IL, USA).

## 3. Results

We have previously shown that Grx2 and Trx1 are induced in the hippocampus upon perinatal asphyxia [23]. Interestingly, Grx2 and Trx1 are essential for the neuronal integrity, since the knock-down of these redoxins in the neuroblastoma cell line SH-SY5Y affected cell morphology and viability during hypoxia and reoxygenation [23]. Within this study, we investigated whether recombinant Grx2 and Trx1 can be used as therapeutics following perinatal asphyxia, using the well-established murine carotid ligation model.

### 3.1. Grx2 Administration Decreases Neuronal Damage after Neonatal Hypoxia/Ischemia

**Figure 1:**
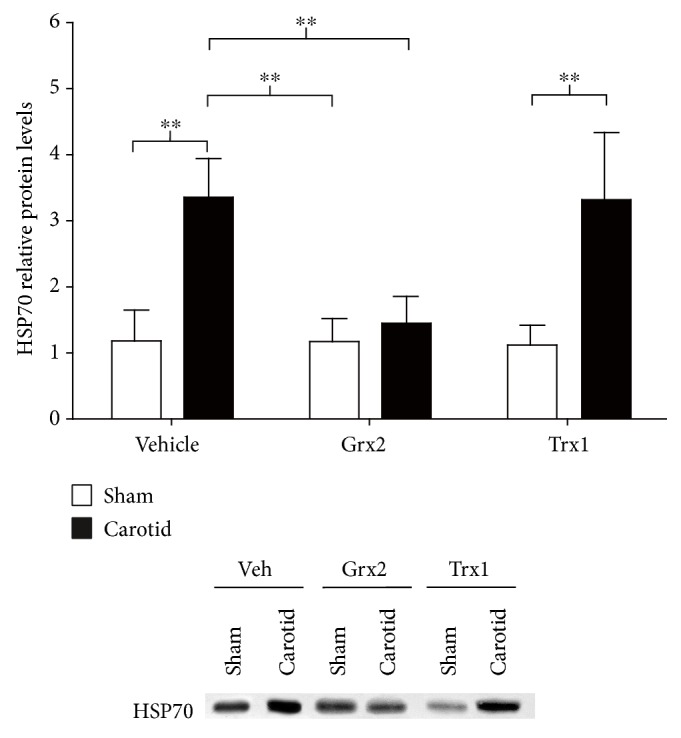
Grx2 reduces neuronal damage following perinatal hypoxia/ischemia. Perinatal asphyxia was induced in rats by carotid ligation and 3 min nitrogen treatment. During 1 h postischemia, the animals were treated with recombinant Grx2 or Trx1. Hippocampi were isolated and analyzed for changes in the protein levels of the neuronal damage marker HSP70 by Western blot. The diagram depicts the relative protein levels compared to sham-operated rats that only received a saline solution (vehicle). Representative Western blots demonstrate the significant changes. Bars depict the mean + SEM of 5 rats per group. Two-way ANOVA test [condition (sham, carotid): *F*_(1, 24)_ = 49.85, *p* < 0.001; treatment (veh, Grx2, and Trx1): *F*_(2, 24)_ = 8.05, *p* < 0.01; and interaction: *F*_(2, 24)_ = 8.39, *p* < 0.01] followed by Tukey's post hoc tests was employed to analyze the data. ^∗∗^*p* < 0.01.

Perinatal asphyxia was induced in rats by carotid ligation and 3 min nitrogen treatment. Following the treatment with recombinantly expressed and purified Grx2, Trx1, or saline solution 1 h after the hypoxic/ischemic event, animals were sacrificed and their brains analyzed. We observed that animals from the carotid ligation group that only received a saline injection displayed an approximately 3-fold higher immunoreactivity against the neuronal damage marker heat shock protein 70 (HSP70) than the sham group with the same treatment (*p* < 0.01) (Figure 1). The carotid ligation group treated with recombinant human Grx2 presented an approximately 3-fold lower immunoreactivity against HSP70 than the carotid ligation group with saline injection (*p* < 0.01) and did not differ from both, the sham group treated with Grx2 nor the sham group treated with saline solution (Figure 1). Treatment with recombinant human Trx1 did not affect the protein levels of HSP70 in both, sham (*p* < 0.01) and carotid ligation animals (*p* < 0.01). The carotid ligation group treated with Trx1 also presented a 3-fold higher immunoreactivity against HSP70, comparable to the sham carotid ligation group (Figure 1).

### 3.2. Exogenous Administration of Grx2 or Trx1 Interferes with the Glutamate Excitotoxicity Pathway after Neonatal Hypoxia/Ischemia

**Figure 2:**
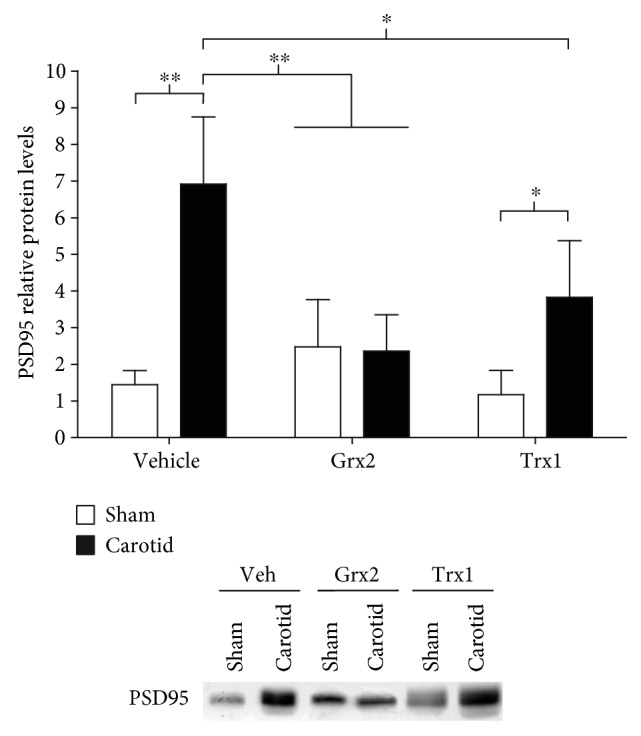
Grx2 and Trx1 interfere with the glutamate excitotoxicity pathway following neonatal hypoxia/ischemia. Perinatal asphyxia was induced in rats by carotid ligation and 3 min nitrogen treatment. During 1 h postischemia, the animals were treated with recombinant Grx2 or Trx1. Hippocampi were isolated and analyzed for changes in the protein levels of PSD95 by Western blot, as a reporter for the induction of glutamate exitotoxicity. The diagram depicts the relative protein levels compared to sham-operated rats that only received a saline solution (vehicle). Bars show the mean + SEM of 5 rats per group. Two-way ANOVA test [condition (sham, carotid): *F*_(1, 24)_ = 29.07, *p* < 0.001; treatment (veh, Grx2, and Trx1): *F*_(2, 24)_ = 58.36, *p* < 0.05; and interaction: *F*_(2, 24)_ = 10.61, *p* < 0.001] followed by Tukey's post hoc tests was employed to analyze the data. ^∗^*p* < 0.05, ^∗∗^*p* < 0.01.

To determine whether Grx2 or Trx1 treatments affect the excitotoxic effects of glutamate associated with PA, we analyzed the protein levels of the postsynaptic density protein 95 (PSD95). High levels of PSD95 have been linked to increase glutamate excitotoxicity, while its reduction has been linked to a neuroprotective effect in neonatal models of hypoxia/ischemia [33–35]. Western blotting analysis showed that animals subjected to neonatal hypoxia/ischemia and injected with saline solution presented over 5-fold higher levels of PSD95 in comparison to the sham group with the same treatment (*p* < 0.01) (Figure 2). After injection of 10 mg/kg of Grx2 1 hour after the hypoxic/ischemic insult, the carotid ligation group displayed no differences compared to the sham group that was also treated with Grx2. Furthermore, neither the sham group nor the carotid group treated with Grx2 showed any significant differences when compared to the sham group that only received the saline injection (Figure 2). The carotid ligation group treated with Grx2 presented an approximately 6-fold reduction in the levels of PSD95 compared with the saline-treated carotid ligation group (*p* < 0.01) (Figure 2). The carotid ligation group treated with Trx1 presented a 3-fold increase in PSD95 levels in comparison to both the sham group treated with Grx2 (*p* < 0.05) and the sham group with saline injection (*p* < 0.05). Nonetheless, Trx1 treatment significantly attenuated the increase in PSD95 levels in the carotid ligation group by 3-fold in comparison to the carotid group treated with saline solution (*p* < 0.01) (Figure 2).

### 3.3. Exogenous Administration of Grx2 or Trx1 Helps in Maintaining Structural Integrity after Neonatal Hypoxia/Ischemia

**Figure 3:**
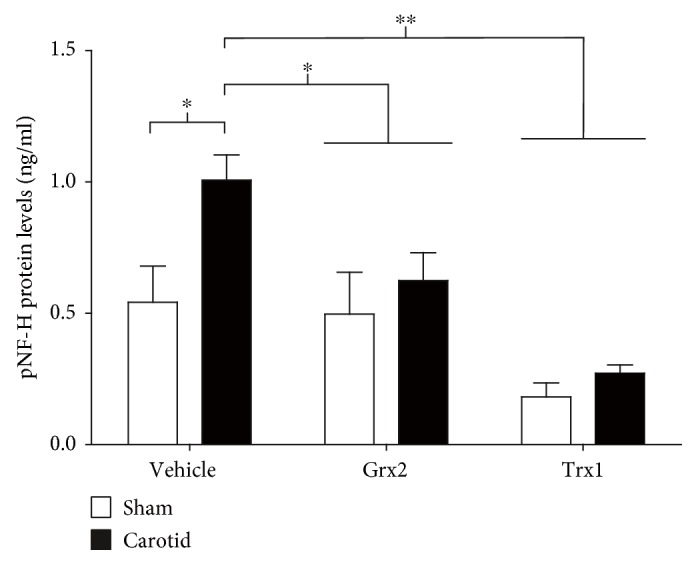
Grx2 and Trx1 help to maintain the structural integrity following neonatal hypoxia/ischemia. Plasma from 10-day-old rats that were subjected to a carotid ligation or sham operation and subsequent 3 min nitrogen treatment and received a treatment with either saline solution, Grx2, or Trx1 one hour postinjury, was screened for pNF-H as a measurement of structural damage to the axons. The levels of pNF-H were analyzed by a specific ELISA. Bars represent the mean + SEM of 5 rats per group. Two-way ANOVA test [condition (sham, carotid): *F*_(1, 24)_ = 27.20, *p* < 0.001; treatment (veh, Grx2, and Trx1): *F*_(2, 24)_ = 53.34, *p* < 0.001; and interaction: *F*_(2, 24)_ = 7.47, *p* < 0.01] followed by Tukey post hoc tests was employed to analyze the data. ^∗^*p* < 0.05, ^∗∗^*p* < 0.01.

The structural integrity of axons was evaluated in hypoxic/ischemic animals 72 h after the hypoxia/ischemia event by analyzing the plasma levels of phosphorylated neurofilament heavy protein (pNF-H). In fact, animals of the CCA ligation group with the saline injection had a 50% increase in the levels of pNF-H in plasma in comparison to the sham group treated only with saline solution (*p* < 0.05) (Figure 3). Animals subjected to an injection of 10 mg/kg of Grx2 one hour after the hypoxic/ischemic event did not show the increase in pNF-H levels in plasma normally associated with structural axon damage. Moreover, both the carotid ligation and sham groups treated with Grx2 did not display differences in the levels of pNF-H, compared to the sham group that only received a saline injection (Figure 3). Similarly, animals from the carotid ligation group treated with Trx1 did not present an increase in pNF-H plasma levels. Interestingly, the levels of pNF-H in plasma in the groups treated with Trx1 were still another 50% lower than those detected in the sham group that only received a saline injection (*p* < 0.05) (Figure 3).

### 3.4. Exogenous Administration of Grx2 and Trx1 Avoids the Astrogliosis Caused by Neonatal Hypoxia/Ischemia

**Figure 4:**
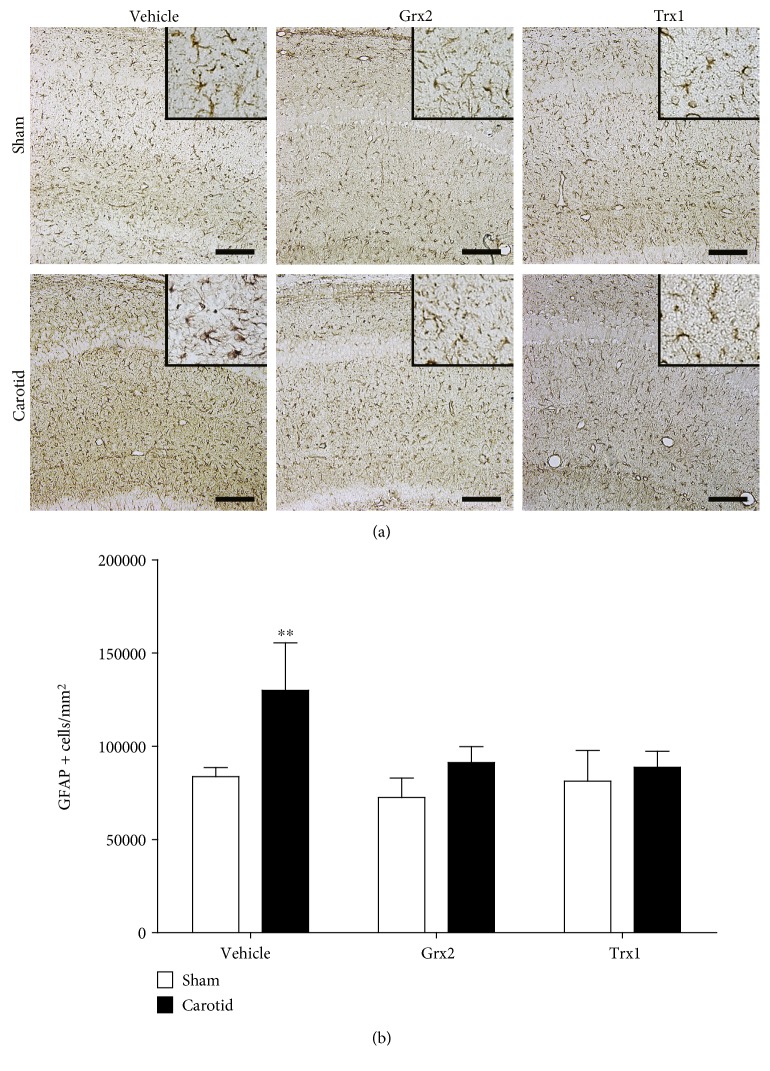
Grx2 and Trx1 prevent the characteristic astrogliosis that follows neonatal hypoxia/ischemia. (a) Representative immunostainings of GFAP in the CA1 area of the hippocampus. Insets show the morphological details of astrocytes. (b) Quantification of GFAP+ cells as number of positive cells by mm^2^ was used as a measurement of the reactive gliosis response. Bars represent the mean + SEM of 4 rats per group. Two-way ANOVA test [condition (sham, carotid): *F*_(1, 18)_ = 17.52, *p* < 0.001; treatment (veh, Grx2, and Trx1): *F*_(2, 18)_ = 7.42, *p* < 0.001; and interaction: *F*_(2, 18)_ = 3.97, *p* < 0.05] followed by Tukey's post hoc tests was employed to analyze the data. ^∗∗^*p* < 0.01. Scale bars 10 *μ*m.

The effect of Grx2 and Trx1 treatment on astrogliosis development following the hypoxic/ischemic event was analyzed by glial fibrillary acidic protein (GFAP) immunostaining in the *stratum radiatum* of the hippocampal CA1 area, which previously has been reported as a particularly susceptible area in neural plasticity and development of astrogliosis [36–38]. As expected, hypoxic/ischemic rats injected with saline solution presented an approximately 30% increase in the number of GFAP positive (GFAP+) astrocytes, in comparison to sham rats treated with saline solution (*p* < 0.01) (Figure 4). Animals from the carotid ligated group treated with Grx2 did not present an increase in the number of GFAP+ cells when compared to the carotid ligated group treated with saline solution (*p* < 0.05), while it did not show any differences in comparison with the sham group treated with Grx2 (Figure 4). Likewise, the carotid group treated with Trx1 did not show an increase in GFAP+ astrocytes in comparison to the carotid group that only received the saline solution injection (*p* < 0.01). No significant difference was detected between the carotid ligation and sham groups treated with Trx1 (Figure 4). Moreover, all groups treated with either Grx2 or Trx1 did not show significant differences when compared with the sham group treated only with the saline solution (Figure 4), suggesting that the treatment with either Grx2 or Trx1 protects from the astrogliosis associated with the hypoxic/ischemic event during birth.

### 3.5. Grx2 and Trx1 Administration Reverts Some of the Early Developmental Alterations Associated with Neonatal Hypoxia/Ischemia

**Figure 5:**
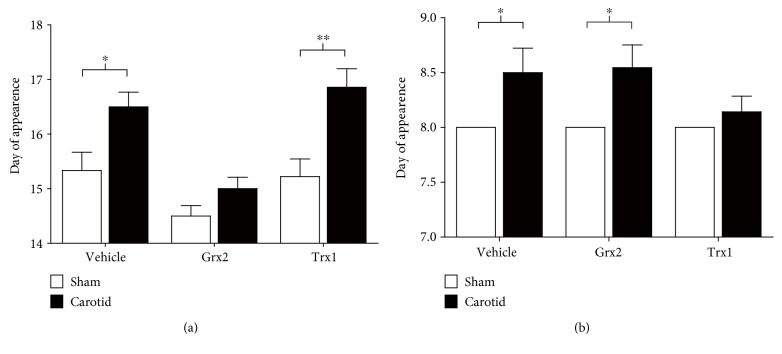
Grx2 and Trx1 revert the delay in the appearance of eye neurodevelopment milestones present after neonatal hypoxia/ischemia. Pups were evaluated for the detection of variation in the appearance of different reflexes and motor skills. (a) Quantification of the eye opening in pups expressed as the day of the first appearance (both eyes presented completed separation of the eyelids). (b) Quantification of the eye twitching reflex in pups expressed as the day of the first appearance (day in which both eyelids twitch for the first time). Bars represent the mean + SEM of 10 rats per group. Kruskal-Wallis tests [eye opening: *H* = 25.35, d.f. = 5, *p* < 0.001; eye twitching: *H* = 15.72, d.f. = 5, *p* < 0.01], followed by post hoc Mann–Whitney tests were employed to analyze the data. ^∗^*p* < 0.05, ^∗∗^*p* < 0.01.

In order to assess both the effects of the hypoxic/ischemic event and the treatment with Grx2 or Trx1 on the neurodevelopment of pups, a series of reflexes and physical developmental parameters were measured from postnatal day 8 (P8) until ~P19. There were no significant differences between group conditions (sham or carotid) or between treatments (saline, Grx2, or Trx1 administration) for the tasks evaluating surface righting reflex, negative geotaxis, crossed extensor reflex, ear twitching, auditory startle, and ear unfolding (see Supplementary Figure 1 available online at https://doi.org/10.1155/2017/4162465). Nevertheless, carotid animals that only received a saline injection displayed a significant delay in eye opening and eyelid twitching in comparison with sham-operated animals (*p* < 0.05 in both cases, see Figures 5(a) and 5(b), resp.). Interestingly, Grx2 administration reverted the delayed eye opening observed in the carotid group treated with saline (Figure 5(a)). Trx1 administration had no effect on this developmental parameter. Interestingly, Grx2 had no effect on the delay in eyelid twitching observed after the injury, while Trx1 treatment reverted the delay in relation with the sham group (Figure 5(b)).

## 4. Discussion

Recently, we have analyzed the changes in the levels of 14 members of the Trx family in the brains of rats subjected to the carotid ligation model [23], showing that Grx2 and Trx1 are crucial for the recovery following hypoxia/ischemia and reoxygenation. In fact, silencing of these proteins in neuron-like cell cultures (SH-SY5Y) plays an important role in maintaining the neuronal phenotype of these cells [23]. These studies prompted us to investigate the effects of protein administration on the neuronal damage caused by PA. The results presented suggest that the administration of Grx2, and to some extent the administration of Trx1, has the potential to significantly attenuate the neuronal damage caused by PA, including the cellular damage response, glutamate excitotoxicity, axonal integrity, and astrogliosis. It is worth mentioning that the reoxygenation phase following a hypoxic/ischemic event comprises the early, acute phase that is induced by O_2_, O_2_−, and overall changes in cellular redox properties, and the late subacute phase that is rather induced by the overall immune response [39]. Here, we have focused on the short-term changes (72 h posthypoxic/ischemic event) induced by PA, because (i) the CNS is especially susceptible to oxidative damage due to its high metabolic rate, its oxygen consumption, and its low capacity for regeneration and (ii) these changes correlate with the mortality rate of patients [40–43].

Interestingly, Prxs, substrates for Trxs and Grxs, are significantly altered as a result of stroke-related insults [21, 44–46]. Overexpression of Prx6 decreased hypoxia-induced retinal ganglion cell death [47]. Prx3, which was shown to be reduced and regenerated by Grx2 [48], showed elevated protein levels in the hippocampus of gerbils following cerebral hypoxia/ischemia and reoxygenation and protected CA1 pyramidal neurons against the induced damage [49]. It is thus tempting to speculate that the protective mechanism of Grx2 could involve the reduction of Prxs and therefore the regulation of intracellular levels of peroxides and cellular signaling, as well as the molecular damage that can occur in the presence of high peroxide levels.

Cellular damage derived from an ischemic insult induces the expression of heat shock proteins, one of the groups of proteins involved in the overall stress response [50]. HSP70 is constitutively expressed and is the most abundant heat shock protein present in the cell, and it is produced as a response to several stimuli, such as heat, heavy metals, toxins, and ischemia [51]. Increased HSP70 expression has been reported as a response to ischemic damage in neurons, astrocytes, and endothelial cells [52]. Interestingly, cerebral ischemia in rats induces HSP70 expression, mainly in the hippocampus and the cerebral cortex, correlating with the vulnerability of the CNS to ischemic damage [53]. Since carotid animals that were treated with Grx2 presented significantly lower levels of HSP70 compared to control animals that only received saline solution, it can be inferred that Grx2 can significantly reduce the immediate cellular response towards the hypoxic/ischemic event associated with the HSP70 response (Figure 1).

The protein-protein interactions, mediated by the PPDZ domain of the postsynaptic density 95 protein, are key elements in intracellular signaling [54]. The structural postsynaptic protein PSD95 binds simultaneously to the ionotropic glutamate receptor NMDA and the enzyme nNOS through its PDZ1 and PDZ2 domains [55]. The activation of the NMDA receptor causes the influx of intracellular Ca^2+^ which, in turn, produces the activation of nNOS with the consequent generation of NO [35], one of the most common promoters of glutamate excitotoxicity [56, 57]. Considering that PSD95 inhibition does not affect the overall ion flux [58] or the signaling pathways that favor survival [59], which are mediated by the NMDA receptor, it has been speculated that PSD95 could be a safe and efficient target for the treatment of cerebral ischemic damage [60]. Hence, neurons deficient for PSD95 or nNOS show a reduction in the vulnerability to glutamate excitotoxicity [61]. Previous studies have shown that the reduction of PSD95 expression is linked to a reduction of the damage associated with a neonatal hypoxic/ischemic event [33–35]. As expected, the levels of PSD95 were significantly elevated in the carotid group compared to the sham animal group (about 7-fold increase). Interestingly, no significant increase of PSD95 was detected in the carotid animals injected with Grx2 compared to the sham animal groups injected with saline solution or Grx2. Thus, Grx2 might not only counteract the HSP70 response but also the damage associated to glutamate excitotoxicity after the hypoxic/ischemic injury (Figure 2). Even though Trx1 treatment did not show a significant effect on HSP70 protein levels following the hypoxia/ischemia (Figure 1), a significant 3-fold decrease in the protein levels of PSD95 compared with the carotid group treated with saline solution was detected. However, Trx1 treatment was not as efficient as Grx2 in preventing or counteracting the induction of PSD95 (Figure 2).

The screening of specific components in tissues and biological fluids secreted upon pathological conditions is commonly used as a therapeutic and diagnostic tool [62]. Neurofilaments are highly abundant proteins in neurons and are found in the axons of all neurons. Their main function is the maintenance of the axonal gauge, which is essential for the morphological integrity and the conduction velocity of neuronal impulses [63]. Three types of neurofilaments (light neurofilaments (NF-L), medium neurofilaments (NF-M), and heavy neurofilaments (NF-H)) make up the nerve fibers and are eliminated to the extracellular space in considerable amounts following axonal damage or neuronal degeneration [64–66]. The perturbation of the axonal membrane expels neurofilaments to the interstitial space and eventually to the cerebrospinal fluid and the blood. In this manner, the levels of neurofilaments in the blood can be used for both the prediction and monitoring of the progress of a disease and the evaluation of the efficacy and/or toxicity of neuroprotective treatments [67–70]. The phosphorylated form of NF-H is axon-specific and is used as a specific marker for neuronal damage and degeneration [71]. pNF-H levels are, for instance, elevated following acute hypoxic/ischemic damage [72]. It has been further shown that the levels of pNF-H in serum and cerebrospinal fluid are increased after a variety of damages to the CNS, both in animal models and in humans [71]. Moreover, the increment of pNF-H levels in serum is a good indicator of the degree of the lesion in the white matter of the CNS [73]. In our model, we could confirm that acute hypoxia/ischemia leads to neuronal damage and elevated levels of pNF-H in serum compared to sham animals (Figure 3). Interestingly, when the carotid animals were treated with Grx2 or Trx1 following the hypoxic/ischemic event, no such increase was detectable via specific ELISA (Figure 3). It is worth to notice that in the case of Trx1 administration, pNF-H levels both in sham and carotid animals were well below those found in the sham group treated only with saline solution. This effect could be the consequence of a higher effect in axonal integrity produced by Trx1. In this regard, previous studies have linked Trx1 to a strong protection against axonal damage in a model of retinal nerve damage [74, 75], as well as to the regulation of axonal regeneration [76]. The excess of Trx1 in the system could thus contribute to lower the basal levels of pNF-H by tipping the balance toward axonal regeneration rather than disruption.

Reactive gliosis can be triggered in response to several CNS pathologies, such as trauma, stroke, and neurodegenerative diseases [77]. Following injury, astrocytes proliferate and their GFAP expression increases [78]. Experimental data suggest that reactive gliosis can be detrimental for neuroplasticity and regeneration of the CNS. Therefore, the prevention of reactive gliosis has become an essential issue in the development of therapeutic interventions [79]. Previous studies have shown that perinatal hypoxia/ischemia triggers reactive gliosis in neonatal rats [36, 80]. In this study, we have shown that hypoxia/ischemia induces the characteristic reactive gliosis. Sham-treated animals, as well as animals that were treated with either Grx2 or Trx1 one hour after the injury, did not develop astrogliosis (Figure 4).

The rat postnatal development is characterized by the appearance of a series of reflexes and motor skills through the first three weeks of life [81]. During this period, different insults, such as a hypoxic/ischemic event, can affect the normal development of those reflexes and physical skills [30, 82–84]. Previous studies have shown that glutamate neurotoxicity plays a key role in the impairment of the development of neurological reflexes and motor skills [82, 85]. This excitotoxicity has shown to be most severe in the retina, in the cortex, and in the hippocampus [82, 85–88]. Moreover, glutamate excitotoxicity has also been previously linked to neurobehavioral delays [82, 85]. Interestingly, the biochemical findings of this study showed that both Grx2 and Trx1 acted in the biomarker reduction associated with the glutamate excitotoxicity pathway which was increased after hypoxia/ischemia (Figure 2). In this regard, neurodevelopmental analyses have shown that Grx2 treatment was able to revert the developmental delay presented in the opening of the eye in carotid pups (Figure 5(a)), while Trx1 administration reverted the neurodevelopmental delay presented in the appearance of the eye twitching reflex (Figure 5(b)).

Both of the thioredoxin family proteins investigated here, Grx2 and Trx1, have been characterized as crucial for cell survival under various conditions when expressed intracellularly (summarized, for instance in Holmgren et al. [89] and Hanschmann et al. [6]). Since Trx1 is known to be secreted by cells via an unconventional mechanism and to function, for instance, in the activation of immune cells (summarized in Nakamura [90]), Trx1 administration has been discussed as a potential therapeutic strategy against cellular damage [91]. In fact, the perfusion, but not the inhalation, of recombinant human Trx1 protected rat lungs from ischemia-reperfusion injury [92]. An intravitreous injection of recombinant Trx1 was also shown to attenuate the damage produced to the retina by ischemia and glaucoma, induced by N-methyl-D-aspartate, which stimulates glutamate receptors [93]. Both Grxs and Trxs have shown to protect from dopamine-induced neuronal damage in vitro [94, 95]. Recently, Tian and coworkers demonstrated that treatment with recombinant Trx1 can improve the neurogenesis via the regulation of the ERK signaling pathway in mice following global cerebral ischemia, improving spatial learning and memory [22].

In the present study, we have shown that even though Grx2 and Trx1 are both part of the thioredoxin family of proteins, they do not necessarily behave in the same manner. This can be exemplified by the differences observed in response to Grx2 and Trx1 treatments, regarding HSP70, PSD95, and pNF-H markers as well as the different outcomes in the neurodevelopmental evaluation after the treatments. These differences could be explained through the possible distinct mechanisms involved in each protein intervention. In this regard, it is known that Grx2, as part of the glutaredoxin subfamily, plays a key role in redox-dependent cellular processes [96]. Particularly, glutaredoxins (Grxs) are able to reduce both disulfides and mixed disulfides, while they can be restored by glutathione reductase which is directly coupled to the GSH/GSSG ratio (the main indicator of the cell redox state and its redox potential) [97, 98]. Grxs are also capable of catalyzing S-glutathionylation of proteins (a key regulatory mechanism of biological processes) [99, 100]. It is generally accepted that Grxs are a key component in response to an oxidative imbalance through the regulation of mix disulfides [101, 102]. In addition, due to the mitochondrial localization of Grx2, it has been proved that it prevents apoptosis by avoiding the leak of cytochrome c from mitochondria and cardiolipin oxidation [89, 103, 104]. By contrast, Trx1 shows no activity towards mixed disulfides and it is thought to have a more prominent role in redox regulation of cell signaling rather than direct participation in redox-dependent reactions as a consequence of its low levels in comparison with other endogenous antioxidants, and its multifunctional role as a cytosolic protein. In this compartment, Trx1 can function as a growth factor and enzyme cofactor and, under different stresses, can translocate to the nucleus and regulate several transcription factors [96, 105–109]. Taking this data into account, the observed differences in the measurements performed in the present study could be associated with the biological processes in which each redoxin is more important. One could hypothesize that those processes more related to an imbalance of the redox state could respond better to a Grx2 treatment, while Trx1 could be more prominent in processes where it functions as a regulator of transcription factors.

## 5. Conclusion

Taken together, the results presented in this study suggest that the delivery of recombinant Grx2, and to some extend of Trx1, has the potential to attenuate the severe neurological damage induced by PA. If not the delivery of these proteins themselves, a detailed understanding of the underlying redox regulated signaling pathways, may put in evidence the need for new therapeutic strategies to improve CNS recovery after PA and potentially other stroke associated pathologies.

## Supplementary Material

Supplementary figure 1: Neurobehavioral development after neonatal hypoxia/ischemia episode and administration of saline solution, Grx2 (10 mg/kg) or Trx1 (10 mg/kg) one hour post-injury. Neurodevelopmental development was evaluated from postnatal day 8 (P8) until ~P19. No statistical differences were detected between the different groups for a) negative geotaxis at P10 (H = 8.82, d.f. = 5, p = n.s.), b) negative geotaxis at P14 (H = 2.31, d.f. = 5, p = n.s.), c) crossed extensor reflex (H = 1.76, d.f. = 5, p = n.s.), d) ear unfolding (H = 3.82, d.f. = 5, p = n.s.), and e) auditory startle (H = 7.66, d.f. = 5, p = n.s.). All animals presented the surface righting reflex and ear twitching at P8 (data not shown). All statistical analyses were performed by Kruskal-Wallis tests. Bars depict the mean + SEM of 10 pups/group. Sham: pups subjected to sham-surgery at P7; Carotid: pups subjected to right common carotid artery ligation followed by 3 min exposure to 100 % nitrogen at P7; Grx2: mouse recombinant glutaredoxin 2 treatment (10 mg/kg) one hour post-injury; Trx1: human recombinant thioredoxin 1 treatment (10 mg/kg) one hour post-injury.
