# Supplementary material for: Neuronal Damage Induced by Perinatal Asphyxia Is Attenuated by Postinjury Glutaredoxin-2 Administration
Source: Oxid Med Cell Longev. 2017 Jun 15;2017:4162465. doi: 10.1155/2017/4162465 (PMC5494587; doi:10.1155/2017/4162465)
Supplement: Supplementary file 1 — Supplementary figure 1: Neurobehavioral development after neonatal hypoxia/ischemia episode and administration of saline solution, Grx2 (10 mg/kg) or Trx1 (10 mg/kg) one hour post-injury. Neurodevelopmental development was evaluated from postnatal day 8 (P8) until ~P19. No statistical differences were detected between the different groups for a) negative geotaxis at P10 (H = 8.82, d.f. = 5, p = n.s.), b) negative geotaxis at P14 (H = 2.31, d.f. = 5, p = n.s.), c) crossed extensor reflex (H = 1.76, d.f. = 5, p = n.s.), d) ear unfolding (H = 3.82, d.f. = 5, p = n.s.), and e) auditory startle (H = 7.66, d.f. = 5, p = n.s.). All animals presented the surface righting reflex and ear twitching at P8 (data not shown). All statistical analyses were performed by Kruskal-Wallis tests. Bars depict the mean + SEM of 10 pups/group. Sham: pups subjected to sham-surgery at P7; Carotid: pups subjected to right common carotid artery ligation followed by 3 min exposure to 100 % nitrogen at P7; Grx2: mouse recombinant glutaredoxin 2 treatment (10 mg/kg) one hour post-injury; Trx1: human recombinant thioredoxin 1 treatment (10 mg/kg) one hour post-injury. [file 4162465.f1.pdf]

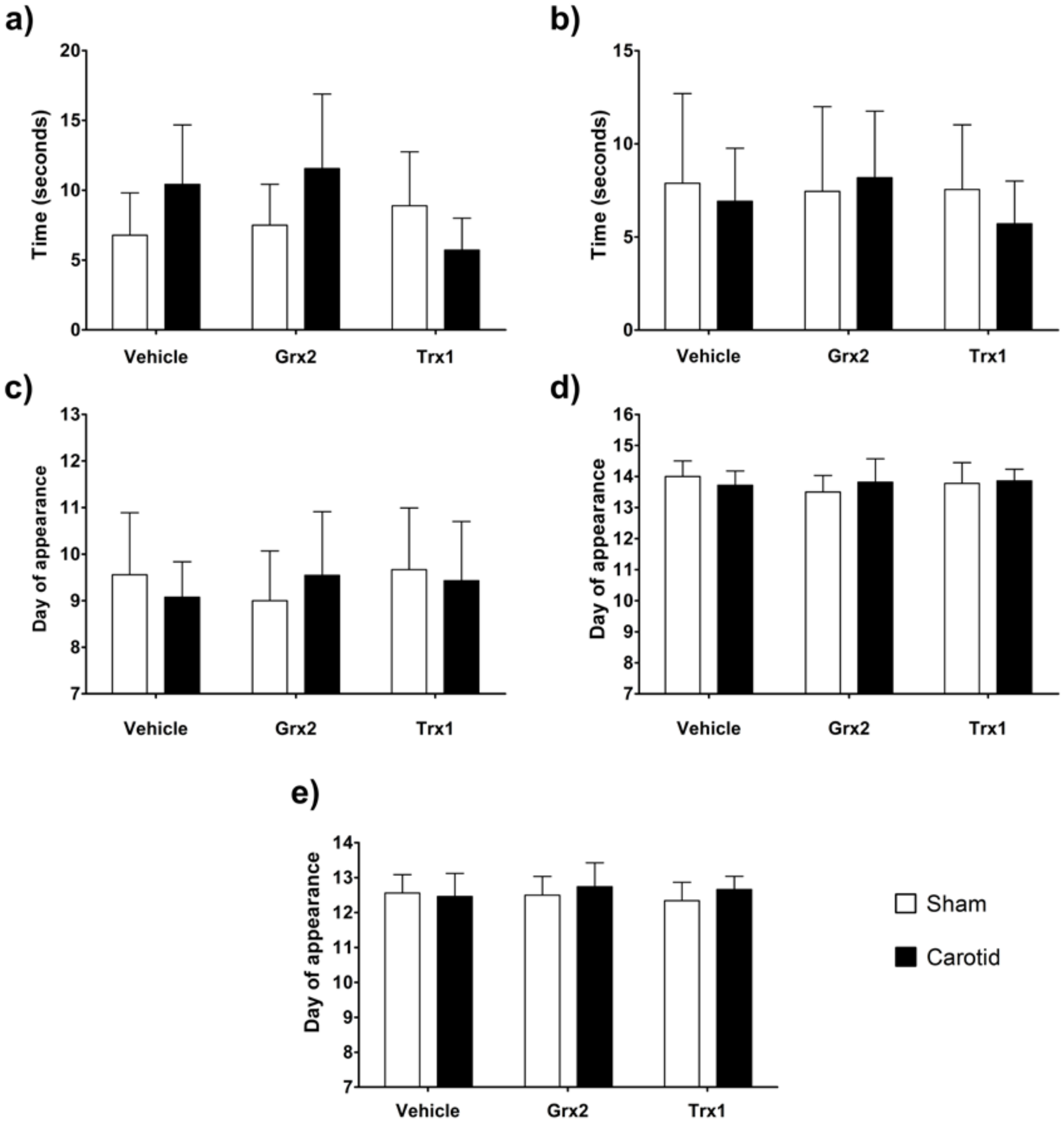

Supplementary figure 1: Neurobehavioral development after neonatal hypoxia/ischemia episode and administration of saline solution, Grx2 (10 mg/kg) or Trx1 (10 mg/kg) one hour post-injury. Neurodevelopmental development was evaluated from postnatal day 8 (P8) until ~P19. No statistical differences were detected between the different groups for **a)** negative geotaxis at P10 (H = 8.82, d.f. = 5, p = n.s.), **b)** negative geotaxis at P14 (H = 2.31, d.f. = 5, p = n.s.), **c)** crossed extensor reflex (H = 1.76, d.f. = 5, p = n.s.), **d)** ear unfolding (H = 3.82, d.f. = 5, p = n.s.), and **e)** auditory startle (H = 7.66, d.f. = 5, p = n.s.). All animals presented the surface righting reflex and ear twitching at P8 (data not shown). All statistical analyses were performed by Kruskal-Wallis tests. Bars depict the mean + SEM of 10 pups/group. Sham: pups subjected to sham-surgery at P7; Carotid: pups subjected to right common carotid artery ligation followed by 3 min exposure to 100 % nitrogen at P7; Grx2: mouse recombinant glutaredoxin 2 treatment (10 mg/kg) one hour post-injury; Trx1: human recombinant thioredoxin 1 treatment (10 mg/kg) one hour post-injury.
